# Cross-metathesis of allylcarboranes with *O*-allylcyclodextrins

**DOI:** 10.3762/bjoc.6.126

**Published:** 2010-11-23

**Authors:** Ivan Šnajdr, Zbyněk Janoušek, Jindřich Jindřich, Martin Kotora

**Affiliations:** 1 Department of Organic and Nuclear Chemistry, Faculty of Science, Charles University in Prague, Hlavova 8, 128 43 Praha 2, Czech Republic; 2 Institute of Inorganic Chemistry of the Academy of Science, v.v.i., Husinec-Řež 1001, 250 68 Řež, Czech Republic

**Keywords:** carborane, catalysis, cross-metathesis, cyclodextrin, ruthenium

## Abstract

Cross-metathesis between allylcarboranes and *O*-allylcyclodextrins was catalyzed by Hoveyda–Grubbs 2^nd^ generation catalyst in toluene. The corresponding carboranyl-cyclodextrin conjugates were isolated in 15–25% yields.

## Introduction

Cross-metathesis of two different alkenes constitutes an efficient and powerful tool for synthesis of various unsymmetrically substituted alkenes. This procedure has found enormous application in organic synthesis of various types of molecules such as natural and biologically active compounds [1]. One of the key aspects of this methodology, which is responsible for a high cross-metathesis selectivity, is a proper choice of a suitable ruthenium catalyst [2–3]. Efficacy of the cross-metathesis procedure has prompted also us to investigate hitherto unexplored combinations of two different alkenes. Recently, we have shown that metathesis of various terminal alkenes with perfluoroalkylpropenes constitutes a simple and efficient approach for the synthesis of wide array of perfluoroalkylated compounds [4]. This methodology was then applied for the synthesis of perfluoroalkylated analogs of brassinosteroids [5], 17α-perfluoroalkylestradiols [6], perfluoroalkylcyclodextrins [7], and perfluoroalkylcarboranes [8]. Successful execution of these reactions prompted us to study also cross-metathesis of allylcarboranes with *O*-allylcyclodextrins as a route to carborane-cyclodextrin conjugates. Herein, we report our preliminary results.

## Results and Discussion

**Figure 1:**
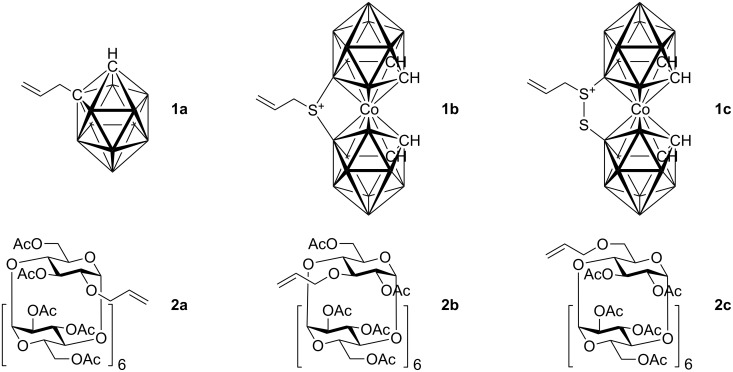
Starting allylcarboranes **1a**–**1c** and *O*-allylcyclodextrins **2a**–**2c**.

Although studies on the inclusion of carboranes into cyclodextrins have previously been reported [9–13], a synthesis of cyclodextrin-carborane conjugates connected by a linker has not been, to the best of our knowledge, described. Since carboranes are of potential interest for various applications in medicine (e.g. boron neutron capture therapy for cancer, radionuclide diagnostics and therapy, and related fields [14–17], whilst some carboranes possess antiviral activity [18–19]), consequently, there is considerable interest in the synthesis of water soluble carborane derivatives. One strategy to access such compounds is based on the synthesis of carborane conjugates bearing a water-soluble moiety. With this in mind, we envisioned that this concept could be realized by the synthesis of carborane-cyclodextrin conjugates by the means of cross-metathesis between readily available allycarboranes and *O*-allylcyclodextrins (Figure 1).

The preparation of the starting allylcarboranes, i.e., 1-allyl-1,2-C_2_B_10_H_11_ **1a** [20], 8,8’-allyl-S-(C_2_B_9_H_11_)_2_Co **1b** [21], and 8,8’-allyl-S_2_-(C_2_B_9_H_11_)_2_Co **1c** [21], was carried out according to the previously reported procedures [8]. *O*-Allylcyclodextrins **2** were prepared by allylation of β-cyclodextrins under various reaction conditions (2^I^-*O*-allyl-β-cyclodextrin for **2a** [22], 3^I^-*O*-allyl-β-cyclodextrin for **2b** [23], and 6^I^-*O*-allyl-β-cyclodextrin for **2c**) followed by peracetylation [7,24].

**Scheme 1:**
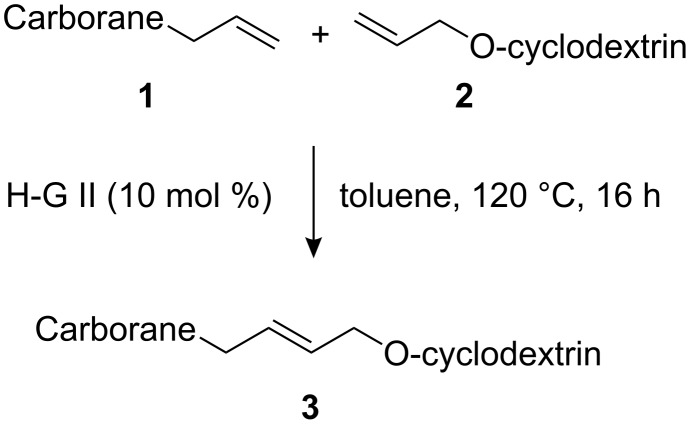
Cross-metathesis of allylcarboranes **1** and *O*-allylcyclodextrins **2**.

At the outset cross-metathesis of allylcarborane **1a** with 2^I^-*O*-allylcyclodextrin **2a** and various ruthenium-carbene complexes (10 mol %) in dichloromethane was carried out to assess the most suitable catalyst (for cross-metatheses involving carboranes, see: [25–26]). However, when the reaction was carried out in the presence of any of the following catalysts, Grubbs 1^st^, Grubbs 2^nd^, or Hoveyda–Grubbs 1^st^ generation, no cross-metathesis products were obtained. Only catalysis by Hoveyda–Grubbs 2^nd^ generation catalyst gave the desired product **3** in 14% yield. The suitability of Hoveyda–Grubbs 2^nd^ generation catalyst for cross-metathesis reactions was consistent with the previously observed results [5–8]. Further tuning of the reaction conditions showed that the best yields were obtained by carrying out the reaction in toluene at 120 °C for 16 h (Scheme 1). Interestingly, when the reactions were carried out in CH_2_Cl_2_ (40 °C) the yields of the corresponding products were lower by 5–10%.

Cross-metatheses of various allylcarboranes **1** and *O*-allylcyclodextrins **2** were then carried out. In general, the reactions proceeded to give the expected products without any problems (Table 1). Thus, the cross-metathesis of **1a** with **2a, 2b,** and **2c** furnished the corresponding carboranylcyclodextrins **3aa**, **3ab**, and **3ac** in 24, 17, and 15% isolated yields, respectively. In an analogous manner the cross-metathesis reactions of **1b** with **2a** and **2c** gave the carboranylcyclodextrins **3ba** and **3bc** in 20 and 19% yields, respectively. Finally, the reactions of the cyclodextrin derivatives **2a**–**2c** with **1c** afforded the corresponding carboranylcyclodextrins **3ca**, **3cb**, and **3cc** in 18, 19 and 20% isolated yields, respectively. It is also of note to mention here the impressive *E*-selectivity of the cross-metathesis reactions, which has also been observed in other metathetical reactions with alkenylcyclodextrins derivatives [27] and can be explained by several factors [28]. Firstly, by a chelation of the intermediate Ru-carbene complex to the oxygen atoms of the cyclodextrin which results in a conformationally rigid intermediate and secondly, by a steric effect of the bulky carborane moiety. Although it may appear that the isolated yields are not high, conversions were in the range of ~50%. Isolation and purification of the products was a tedious task and the isolated yields we obtained represent the amounts of analytically pure compounds. It has been reported that low yields and conversions could be explained by isomerization of terminal to internal double bonds in both reactants (e.g., isomerization of allyl ethers to vinyl ethers [29–31] and allylcarboranes to propenylcarboranes [25]) and thus decreasing the reactant activity. However, NMR analysis of compounds isolated from the reaction mixtures revealed only the presence of the starting material and products, thus the low conversions could be attributed to deactivation of the catalysts by other routes. A similar effect has been also been observed in other cross-metathesis of various *O*-alkenylcyclodextrins which required the use of large amounts of catalyst [32–33]. Attempts to carry out the reaction with free (unprotected) *O*-allylcyclodextrins in dichloromethane or toluene has not so far resulted in the formation of any of the expected products, presumably because of their insolubility in the aforementioned solvents. To overcome the problem of the solubility of free *O*-allylcyclodextrins, the reaction was carried out in water in the presence of surfactant (SDS – sodium dodecyl sulfate), however, cross-metathesis did not occur.

**Table 1:** The prepared carborane-cyclodextrin conjugates **3**.

Reactants		Product		Yield (%)^a^

**1a**	**2a**	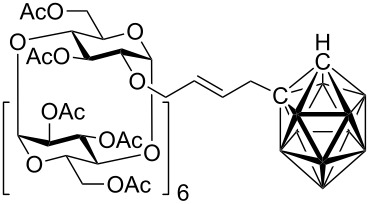	**3aa**	24
	**2b**	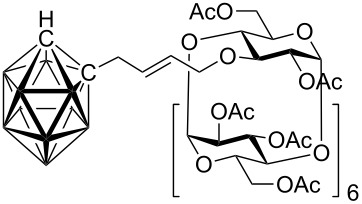	**3ab**	17
	**2c**	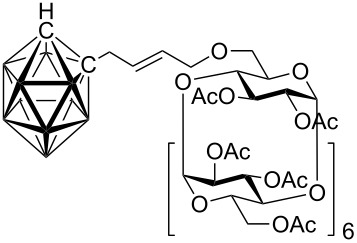	**3ac**	15
**1b**	**2a**	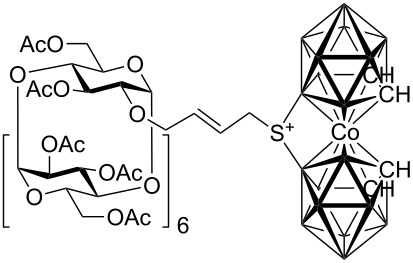	**3ba**	20
	**2c**	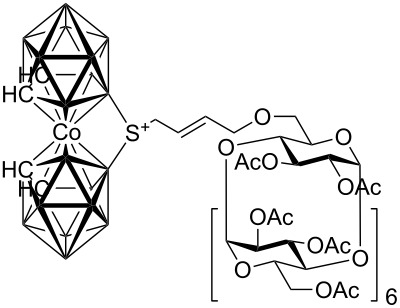	**3bc**	19
**1c**	**2a**	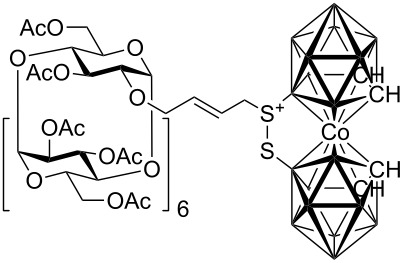	**3ca**	18
	**2b**	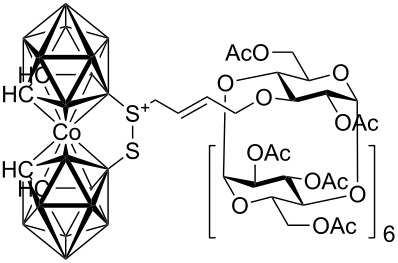	**3cb**	19
	**2c**	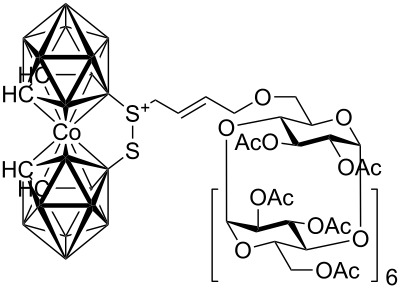	**3cc**	20

^a^Isolated yields.

## Conclusion

The results described above clearly indicate that the cross-metathesis of allylcarboranes and *O*-allylcyclodextrins catalyzed by Hoveyda–Grubbs 2^nd^ generation catalyst provides a simple and straightforward method for the synthesis of cyclodextrin-carborane conjugates. The high boron content and the presence of a water soluble moiety (after removal of the protecting groups) suggest that the compounds may have potential for use in medical applications.

## Experimental

**General procedure for metathesis of allylcyclodextrins with allylcarboranes.** The Hoveyda–Grubbs 2^nd^ generation catalyst (3.13 mg, 0.005 mmol) was added under an argon atmosphere to a mixture of an allylcyclodextrin (0.07 mmol) and an allylcarborane (0.05 mmol) in toluene (5 mL). The resulting solution was stirred at 110 °C overnight. Removal of the solvent under reduced pressure gave a brown residue that was purified by column chromatography (85/15 MeOH/H_2_O) on C_18_-reversed phase.

**Per-*O*-acetyl-2^I^-*O*-[4-(1,2-dicarbadodecaboran-1-yl)-but-2-en-1-yl]-β-cyclodextrin (3aa).** The compound was prepared from **2a** (0.15 g, 0.07 mmol) and **1a** (15 mg, 0.05 mmol). Column chromatography gave the title compound, 0.041 g (24%), as a white powder: m. p. 188–190 °C; IR (KBr) 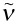 = 2591, 1747, 1371, 1236, 1044 cm^−1^; ^1^H NMR (300 MHz, CDCl_3_): δ = 6.08–6.02 (m, 1 H, H-2’), 5.66–5.58 (m, 1 H, H-3’), 5.46–5.15 (m, 7 H, 7 × H-3), 5.13–4.92 (m, 7 H, 7 × H-1), 4.86–4.70 (m, 6 H, 6 × H-2), 4.61–4.43 (m, 6 H, 6 × H-6), 4.39–3.82 (m, 17 H, 8 × H-6, 7 × H-5, 2 × H-1’), 3.78–3.53 (m, 9 H, 7 × H-4, 2 × H-4’), 3.34 (d, *J* = 7.0 Hz, 1 H, H-2^I^), 2.97 (s, 1 H, C_carb_-H), 2.11–1.97 (m, 60 H, 20 × CH_3_); ^13^C NMR (100 MHz, CDCl_3_): *δ* = 171.15–169.71 (20 × C=O), 132.28 (C-2’), 126.69 (C-3’), 98.35–97.06 (7 × C-1), 78.05–76.42 (7 × C-4), 74.19 (C-1’), 72.29–69.66 (7 × C-2, 7 × C-3, 7 × C-5), 63.10–62.85 (7 × C-6), 60.34 (2 × C_carb_), 40.54 (C-4’), 21.56-21.02 (20 × CH_3_); ^11^B NMR (128 MHz, CDCl_3_): *δ* = −3.29 (s, 1 B, B-9), −6.45 (s, 1 B, B-12), −10.14 (s, 2 B, B-8, B-10), −12.21 (s, 2 B, B-4, B-5), −13.47 (s, 4 B, B-3, B-6, B-7, B-11); MS (EI, *m/z* (rel.%)) 1108.7 (80), 1010.5 (19), 799.0 (12), 516.9 (14), 456.8 (25), 374.0 (29), 242.3 (100), 228.6 (56), 168.9 (41); HR-MS (ESI) calcd. for C_88_H_126_O_55_B_10_: 1108.3925, found 1108.3954 (C_88_H_126_O_55_^10^B_2_B_8_Na_2_).

**Per-*O*-acetyl-3^I^-*O*-[4-(1,2-dicarbadodecaboran-1-yl)-but-2-en-1-yl]-β-cyclodextrin (3ab).** The compound was prepared from **2b** (0.15 g, 0.07 mmol) and **1a** (15 mg, 0.05 mmol). Column chromatography gave the title compound, 0.029 g (17%), as a white powder: m. p. 198–201 °C; IR (KBr): 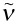 = 2593, 1747, 1369, 1237, 1043 cm^−1^; ^1^H NMR (400 MHz, CDCl_3_): δ = 5.91–5.70 (m, 1 H, H-2’), 5.60–5.50 (m, 1 H, H-3’), 5.49–5.28 (m, 6 H, H-3), 5.24–5.05 (m, 7 H, H-1), 4.89–4.66 (m, 8 H, 7 × H-4, 1 × H-1’), 4.66–3.64 (m, 31 H, 14 × H-6, 1 × H1’, 2 × H-4’, 7 × H-5, 7 × H-4), 3.23–3.13 (m, 1 H, H-3^I^), 2.84 (bs, 1 H, C_carb_-H), 2.20–1.95 (m, 60 H, 20 × CH_3_); ^13^C NMR (100 MHz, CDCl_3_): *δ* = 170.76–170.04 (20 × C=O), 133.74 (C-2’), 126.51 (C-3’), 97.41–96.70 (7 × C-1), 77.68–75.86 (7 × C-4), 72.42–69.46 (7 × C-2, 7 × C-3, 7 × C-5), 62.91–62.18 (7 × C-6), 60.57 (2 × C_carb_), 40.31(C-4’), 21.14–20.87 (20 × CH_3_); ^11^B NMR (128 MHz, CDCl_3_): *δ* = −3.32 (s, 1 B, B-9), −6.47 (s, 1 B, B-12), −10.12 (s, 2 B, B-8, B-10), −12.02 (s, 2 B, B-4, B-5), −13.99 (s, 4 B, B-3, B-6, B-7, B-11); MS (EI, *m/z* (rel.%)): 1108.6 (100), 917.4 (19), 802.8 (20), 690.9 (18), 469.4 (42), 413.2 (65), 370.1 (96), 301.1 (75); HR-MS (ESI) calcd. for C_88_H_126_O_55_B_10_ 1108.3925, found 1108.3957 (C_88_H_126_O_55_^10^B_2_B_8_Na_2_).

**Per-*O*-acetyl-6^I^-*O*-[4-(1,2-dicarbadodecaboran-1-yl)-but-2-en-1-yl]-β-cyclodextrin (3ac).** The compound was prepared from **2c** (0.15 g, 0.07 mmol) and **1a** (15 mg, 0.05 mmol). Column chromatography gave the title compound,0.026 g (15%), as a white powder: m. p. 194–197 °C; IR (KBr): 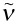 = 2917, 2848, 1747, 1370, 1235, 1044 cm^−1^; ^1^H NMR (400 MHz, CDCl_3_): δ = 5.91–5.82 (m, 1 H, H-2’), 5.75–5.65 (m, 1 H, H-3’), 5.42–5.00 (m, 14 H, 7 × H-3, 7 × H-1), 4.91–4.68 (m, 7 H, 7 × H-2), 4.64–4.42 (m, 5 H, 5 × H-6), 4.40–3.94 (m, 17 H, 2 × H-1’, 5 × H-5, 8 × H-6, 2 × H-4’), 3.90–3.82 (m, 2 H, 2 × H-5), 3.80–3.56 (m, 8 H, 1 × H-6^I^, 7 × H-4), 2.96 (bs, 1 H, C_carb_-H), 2.17–1.97 (m, 60 H, 20 × CH_3_); ^13^C NMR (100 MHz, CDCl_3_): *δ* = 171.10–169.81 (20 × C=O), 134.05 (C-2’), 126.31 (C-3’), 97.45–96.64 (7 × C-1), 77.69–76.30 (7 × C-4), 71.94–69.79 (7 × C-2, 7 × C-3, 7 × C-5, C-1’), 68.12 (C-6^I^), 63.02–62.71 (6 × C-6), 60.18 (2 × C_carb_), 40.08 (C-4’), 21.87–21.16 (20 × CH_3_); ^11^B NMR (128 MHz, CDCl_3_): *δ* = −3.27 (s, 1 B, B-9), −6.69 (s, 1 B, B-12), −10.12 (s, 2 B, B-8, B-10), −12.04 (s, 2 B, B-4, B-5), −13.94 (s, 4 B, B-3, B-6, B-7, B-11); MS (EI, *m/z* (rel.%)): 1108.5 (52), 1010.1 (62), 928.6 (51), 909.0 (60), 667.1 (31), 351.3 (57), 307.3 (100); HR-MS (ESI) calcd. for C_88_H_126_O_55_B_10_: 1108.3925, found 1108.3955 (C_88_H_126_O_55_^10^B_2_B_8_Na_2_).

**Per-*O*-acetyl-2^I^-*O*-{4-{8,8’-μ-(sulfido)-[3,3’-commo-cobalt(III)-bis-(1,2-dicarbaundecaborate)]-8-yl}but-2-en-1-yl}-β-cyclodextrin (3ba).** The compound was prepared from **2a** (0.15 g, 0.07 mmol) and **1b** (20 mg, 0.05 mmol). Column chromatography gave the title compound, 0.023 g (18%), as an orange powder: m. p. 207–209 °C; IR (KBr): 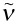 = 2575, 1746, 1371, 1236, 1044 cm^−1^. ^1^H NMR (600 MHz, CDCl_3_): δ = 5.75 (dt, *J* = 20.4, 5.4 Hz, 1 H, H-2’), 5.65–5.58 (m, 1 H, H-3’), 5.43–5.13 (m, 7 H, 7 × H-3), 5.12–4.91 (m, 6 H, 6 × H-1), 4.86 (d, *J* = 3.6 Hz, 1 H, H-1^I^), 4.98–4.62 (m, 6 H, 6 × H-2), 4.56–4.38 (m, 6 H, 6 × H-6), 4.30–3.87 (m, 17 H, 8 × H-6, 7 × H-5, 2 × H-1’), 3.72–3.50 (m, 9 H, 7 × H-4, 2 × H-4’), 3.46–3.34 (m, 4 H, C_carb_-H), 3.24 (dd, *J* = 3.0, 9.6 Hz, 1 H, H-2^I^), 2.09–1.97 (m, 60 H, 20 × CH_3_); ^13^C NMR (150 MHz, CDCl_3_): *δ* = 170.81–169.37 (20 × C=O), 134.55 (C-2’), 122.80 (C-3’), 98.16–96.58 (7 × C-1), 78.05–76.17 (7 × C-4), 71.03–69.54 (7 × C-2, 7 × C-3, 7 × C-5, C-1’), 62.85–62.41 (7 × C-6), 49.33–48.66 (4 × C_carb_), 42.02 (C-4’), 20.99–20.22 (20 × CH_3_); ^11^B NMR (128 MHz, CDCl_3_): *δ* = 1.26 (bs, 2 B, B-8, B-8’), −4.70 (bs, 2 B, B-10, B-10’), −(6.00–11.72) (m, 12 B, B-4, B-4’, B-5, B-5’, B-7, B-7’, B-9, B-9’, B-11, B-11’, B-12, B-12’), −14.68 (bs, 2 B, B-6, B-6’); MS (EI, *m/z* (rel.%)): 1214.4 (100), 1010.3 (10), 413.3 (68), 391.3 (10), 307.2 (52); HR-MS (ESI) calcd. for C_90_H_135_O_55_^10^B_3_B_15_CoNa_2_S: 1213.9194, found 1213.9233.

**Per-*O*-acetyl-6^I^-*O*-{4-{8,8’-μ-(sulfido)-[3,3’-commo-cobalt(III)-bis-(1,2-dicarbaundecaborate)]-8-yl}but-2-en-1-yl}-β-cyclodextrin (3bc).** The compound was prepared from **2c** (0.15 g, 0.07 mmol) and **1b** (20 mg, 0.05 mmol). Column chromatography gave the title compound, 0.028 g (22%), as an orange powder: m. p. 188–191 °C; IR (KBr): 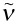 = 2955, 2575, 1747, 1370, 1237, 1046 cm^−1^; ^1^H NMR (600 MHz, CDCl_3_): δ = 5.90 (dt, *J* = 15.1, 5.1 Hz, 1 H, H-2’), 5.78–5.68 (m, 1 H, H-3’), 5.40–5.19 (m, 7 H, 7 × H-3), 5.17–5.02 (m, 7 H, 7 × H-1), 4.86–4.69 (m, 7 H, 7 × H-2), 4.63–4.42 (m, 5 H, 5 × H-6), 4.36–3.96 (m, 17 H, 2 × H-1’, 2 × H-4’, 5 × H-5, 8 × H-6), 3.90–3.85 (m, 2 H, 2 × H-5), 3.78–3.65 (m, 10 H, 1 × H-6^I^, 7 × H-4, 2 × C_carb_-H), 3.48 (bs, 1 H, C_carb_-H), 3.42 (bs, 1 H, C_carb_-H), 2.16–1.98 (m, 60 H, 20 × CH_3_); ^13^C NMR (100 MHz, CDCl_3_): *δ* = 171.10–169.80 (20 × C=O), 134.85 (C-2’), 122.80 (C-3’), 97.46–96.77 (7 × C-1), 77.69–76.21 (7 × C-4), 72.02–69.63 (7 × C-2, 7 × C-3, 7 × C-5, C-1’), 68.28 (C-6^I^), 62.99–62.77 (6 × C-6), 49.93–49.27 (4 × C_carb_), 42.71 (C-4’), 21.15–20.58 (20 × CH_3_); ^11^B NMR (128 MHz, CDCl_3_): *δ* = 1.39 (bs, 2 B, B-8, B-8’), −4.58 (bs, 2 B, B-10, B-10’), −(5.84–11.21) (m, 12 B, B-4, B-4’, B-5, B-5’, B-7, B-7’, B-9, B-9’, B-11, B-11’, B-12, B-12’), −14.92 (bs, 2 B, B-6, B-6’); MS (EI, *m/z* (rel.%)): 1214.1 (100), 1010.3 (10), 414.3 (52), 360.3 (10), 307.2 (96); HR-MS (ESI) calcd. for C_90_H_135_O_55_^10^B_3_B_15_CoNa_2_S: 1213.9194, found 1213.9234.

**Per-*O*-acetyl-2^I^-*O*-{4-{8,8’-μ-(disulfido)-[3,3’-commo-cobalt(III)-bis-(1,2-dicarbaundecaborate)]-8-yl}but-2-en-1-yl}-β-cyclodextrin (3ca).** The compound was prepared from **2a** (0.15 g, 0.07 mmol) and **1c** (20 mg, 0.05 mmol). Column chromatography gave the title compound, 0.026 g (20%), as a red powder: m. p. 188–191 °C; IR (KBr): 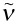 = 2581, 1746, 1371, 1236, 1043 cm^−1^; ^1^H NMR (600 MHz, CDCl_3_): δ = 6.04–5.95 (m, 1 H, H-2’), 5.72–5.63 (m, 1 H, H-3’), 5.34–5.16 (m, 7 H, 7 × H-3), 5.10–4.97 (m, 6 H, 6 × H-1), 4.96 (t, *J* = 3.6 Hz, 1 H, H-1^I^), 4.84–4.76 (m, 6 H, 6 × H-2), 4.58–4.47 (m, 7 H, 7 × H-6), 4.37–4.00 (m, 18 H, 2 × H-1’, 2 × H-4’, 7 × H-5, 7 × H-6), 3.74–3.55 (m, 8 H, 6 × H-4, 2 × C_carb_-H), 3.51–3.45 (m, 1 H, C_carb_-H), 3.37–3.31 (m, 2 H, 1 × H-2^I^, 1 × C_carb_-H), 2.12–1.95 (m, 60 H, 20 × CH_3_); ^13^C NMR (150 MHz, CDCl_3_): *δ* = 171.11–169.56 (20 × C=O), 138.15 (C-2’), 121.0 (C-3’), 98.27–96.81 (7 × C-1), 77.97–76.38 (7 × C-4), 72.24–69.43 (7 × C-2, 7 × C-3, 7 × C-5, C-1’), 63.06–62.62 (7 × C-6), 51.99 (C_carb_), 50.92 (C_carb_), 50.85 (C_carb_), 49.36–49.08 (C-4’, C_carb_), 21.24–21.01 (20 × CH_3_); ^11^B NMR (128 MHz, CDCl_3_): *δ* = 21.13 (bs, 2 B, B-8, B-8’), 0.56 (bs, 2 B, B-10, B-10’), −(1.91–10.86) (m, 12 B, B-4, B-4’, B-5, B-5’, B-7, B-7’, B-9, B-9’, B-11, B-11’, B-12, B-12’), −15.30 (bs, 2 B, B-6, B-6’); MS (EI, *m/z* (rel.%)): 2436.4 (60), 2037.3 (10), 1272.7 (10), 1230.2 (100), 1030.6 (16), 1010.1 (10), 307.1 (61); HR-MS (ESI) calcd. for C_90_H_135_O_55_^10^B_6_B_12_CoNa_2_S_2_: 1228.4109, found 1228.4128.

**Per-*O*-acetyl-3^I^-*O*-{4-{8,8’-μ-(disulfido)-[3,3’-commo-cobalt(III)-bis-(1,2-dicarbaundecaborate)]-8-yl}but-2-en-1-yl}-β-cyclodextrin (3cb).** The compound was prepared from **2b** (0.15 g, 0.07 mmol) and **1c** (20 mg, 0.05 mmol). Column chromatography gave the title compound, 0.029 g (25%), as a red powder: m. p. 201–203 °C; IR (KBr): 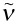 = 2955, 2582, 1747, 1368, 1236, 1042 cm^−1^; ^1^H NMR (600 MHz, CDCl_3_): δ = 6.15–6.06 (m, 1 H, H-2’), 5.74–5.65 (m, 1 H, H-3’), 5.50–5.22 (m, 7 H, 7 × H-3), 5.15–4.99 (m, 6 H, 6 × H-1), 4.85–4.61 (m, 8 H, 7 × H-4, 1 × H-1’), 4.60–3.85 (m, 25 H, 14 × H-6, 2 × H1’, 2 × H-4’, 7 × H-5), 3.84–3.49 (m, 9 H, 7 × H-4, 2 × C_carb_-H), 3.37 (bs, 1 H, C_carb_-H), 3.34 (bs, 1 H, C_carb_-H), 2.12–1.93 (m, 60 H, 20 × CH_3_); ^13^C NMR (150 MHz, CDCl_3_): *δ* = 170.86–169.86 (20 × C=O), 139.90 (C-2’), 119.35 (C-3’), 97.80–96.37 (7 × C-1), 77.89–75.67 (7 × C-4), 73.50 (C-1’), 72.23–69.05 (7 × C-2, 7 × C-3, 7 × C-5), 62.94–62.28 (7xC-6), 51.82 (C_carb_), 51.07 (C_carb_), 50.83 (C_carb_), 49.63–49.42 (C-4’, C_carb_), 21.09–21.00 (20 × CH_3_); ^11^B NMR (128 MHz, CDCl_3_): *δ* = 20.96 (bs, 2 B, B-8, B-8’), 0.54 (bs, 2 B, B-10, B-10’), −(1.99–10.87) (m, 12 B, B-4, B-4’, B-5, B-5’, B-7, B-7’, B-9, B-9’, B-11, B-11’, B-12, B-12’), −15.42 (bs, 2 B, B-6, B-6’); MS (EI, *m/z* (rel.%)): 2438.8 (41), 2250.9 (10), 2037.7 (15), 1229.9 (50), 1030.8 (33), 307.2 (100); HR-MS (ESI) calcd. for C_90_H_135_O_55_^10^B_6_B_12_CoNa_2_S_2_: 1228.4109, found 1228.4127.

**Per-*O*-acetyl-6^I^-*O*-{4-{8,8’-μ-(disulfido)-[3,3’-commo-cobalt(III)-bis-(1,2-dicarbaundecaborate)]-8-yl}but-2-en-1-yl}-β-cyclodextrin (3cc).** The compound was prepared from **2c** (0.15 g, 0.07 mmol) and **1c** (20 mg, 0.05 mmol). Column chromatography gave the title compound, 0.022 g (19%), as a red powder: m. p. 198–200 °C; IR (KBr): 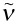 = 2578, 1747, 1370, 1236, 1045 cm^−1^; ^1^H NMR (600 MHz, CDCl_3_): δ = 6.07 (dt, *J* = 15.0, 5.0 Hz, 1 H, H-2’), 5.77–5.70 (m, 1 H, H-3’), 5.40–5.21 (m, 7 H, 7 × H-3), 5.16–5.05 (m, 7 H, 7 × H-1), 4.86–4.69 (m, 7 H, 7 × H-2), 4.63–4.52 (m, 4 H, 4 × H-6), 4.46 (dd, *J* = 10.8, 3.5 Hz, 1 H, 1 × H-6), 4.41–4.03 (m, 17 H, 2 × H-1’, 2 × H-4’, 5 × H-5, 8 × H-6), 3.93–3.89 (m, 2 H, 2 × H-5), 3.87 (m, 2 H, 2 × H-4), 3.75–3.62 (m, 8 H, 1 × H-6^I^, 5 × H-4, 2 × C_carb_-H), 3.49 (bs, 1 H, C_carb_-H), 3.34 (bs, 1 H, C_carb_-H), 2.16–2.00 (m, 60 H, 20 × CH_3_); ^13^C NMR (150 MHz, CDCl_3_): *δ* = 171.13–169.51 (20 × C=O), 137.95 (C-2’), 120.88 (C-3’), 97.38–96.55 (7 × C-1), 77.82–76.21 (7 × C-4), 71.86–68.37 (7 × C-2, 7 × C-3, 7 × C-5, C-1’), 68.37 (C-6^I^), 62.93–62.65 (6 × C-6), 52.23 (C_carb_), 51.68 (C_carb_), 50.99 (C_carb_), 49.64–49.21 (C-4’, C_carb_), 21.07–20.98 (20 × CH_3_); ^11^B NMR (128 MHz, CDCl_3_) *δ* 21.66 (bs, 2 B, B-8, B-8’), 0.18 (s, 1 B, B-10 or B-10’), −1.02 (s, 1 B, B-10 or B-10’), −(1.93-10.95) (m, 12 B, B-4, B-4’, B-5, B-5’, B-7, B-7’, B-9, B-9’, B-11, B-11’, B-12, B-12’), −15.63 (s, 2 B, B-6, B-6’); MS (EI, *m/z* (rel.%)): 2437.8 (23), 2085.6 (11), 1229.9 (20), 1010.1 (15), 528.1 (10), 307.2 (100); HR-MS (ESI) calcd. for C_90_H_135_O_55_^10^B_6_B_12_CoNa_2_S_2_: 1228.4109, found 1228.4119.

## Supporting Information

File 1 Experimental details and characterisation data of peracetylated cyclodextrins **2a**, **2b** and **2c**.
