# Supplementary material for: Cross-metathesis of allylcarboranes with O-allylcyclodextrins
Source: Beilstein J Org Chem. 2010 Nov 23;6:1099–105. doi: 10.3762/bjoc.6.126 (PMC3001993; doi:10.3762/bjoc.6.126)
Supplement: File 1 — Experimental details and characterisation data of peracetylated cyclodextrins 2a, 2b and 2c. [file Beilstein_J_Org_Chem-06-1099-s001.pdf]

Supporting Information

for

Cross-metathesis of allylcarboranes with *O*-allylcyclodextrins

Ivan Šnajdr[1], Zbyněk Janoušek[2], Jindřich Jindřich[1], and Martin Kotora*[1]

Address: [1]Department of Organic and Nuclear Chemistry, Faculty of Science, Charles University in Prague, Hlavova 8, 128 43 Praha 2, Czech Republic and [2]Institute of Inorganic Chemistry of the Academy of Science, v.v.i., Husinec-Řež 1001, 250 68 Řež, Czech Republic.

Email: Martin Kotora - kotora@natur.cuni.cz

* Corresponding author

Experimental details and characterisation data of peracetylated cyclodextrins 2a, 2b and 2c.

General

2^{I}-*O*-allyl-β-cyclodextrin [1], and 3^{I}-*O*-allyl-β-cyclodextrin [2], and 6^{I}-*O*-allyl-β-cyclodextrin [3], were prepared according to the previously described procedures.

General Procedure for Peracetylation of Allylcyclodextrins.

Suspension of allylcyclodextrin (1 mmol) in acetic anhydride (0.47 mol) and triethylamine (0.36 mol) was stirred at 80 °C for 5 hours. After cooling to room

temperature, the reaction mixture was diluted by the addition of 0.5 M HCl (60 mL) and the extracted with $CHCl_3$ (3 × 50 mL). The combined organic extracts were dried over anhydrous Na_2SO_4, concentrated in vacuo to give brown residue that was subsequently purified by column chromatography (silica gel, eluent: $CHCl_3$/MeOH, 100:1.)

Per-*O*-acetyl-2^{I}-*O*-allyl-β-cyclodextrin 2a. The compound was prepared from 2^{I}-*O*-allyl-β-cyclodextrin (1.92 g, 1.6 mmol). Column chromatography gave the title compound, 3.08 g (94%), as a white powder whose spectral data were in agreement with the published data [4].

Per-*O*-acetyl-3^{I}-*O*-allyl-β-cyclodextrin 2b: The compound was prepared from 3^{I}-*O*-allyl-β-cyclodextrin (0.48 g, 0.4 mmol). Column chromatography gave the title compound, 0.77 g (94%), as a white powder: m. p. 130.6 °C; $[\alpha]_D^{20}$ = + 114.2 (c = 0.0026 in MeOH); IR (KBr): $\tilde{\nu}$ = 2953, 1747, 1368, 1237, 1044 cm^{-1}; ^{1}H NMR (600 MHz, $CDCl_3$): δ = 5.91 (ddt, *J* = 17.2, 10.2, 5.4 Hz, 1 H, H-2'), 5.47 (dd, *J* = 9.0, 1.2 Hz, 1 H, H-3), 5.35-5.23 (m, 6 H, 5 × H-3, H-3'b), 5.14–5.10 (m, 2 H, H-3'a, H-1), 5.08–5.01 (m, 6 H, 6 x H-1), 4.80–4.66 (m, 7 H, 7 x H-2), 4.58 (dd, *J* = 12.6, 1.2 Hz, 1 H, H-6), 4.55–4.45 (m, 6 H, 5 × H-6, H-1'a), 4.36 (dd, *J* = 12.6, 4.2 Hz, 1 H, H-6'), 4.29–4.02 (m, 14 H, H-1'b, 7 × H-6, 6 × H-5), 3.91–3.88 (m, 1 H, H-5'), 3.79 (dd, *J* = 9.0, 1.2 Hz, 1 H, H-3^{I}), 3.76–3.64 (m, 6 H, 6 × H-4), 3.56 (t, *J* = 9.0 Hz, 1 H, H-4^{I}), 2.12–1.97 (m, 60 H, 20 x CH_3); ^{13}C NMR (150 MHz, $CDCl_3$): δ = 170.87–169.31 (20 × C=O), 135.49 (C-2'), 115.88 (C-3'), 97.58 (C-1^{I}), 97.35 (C-1), 97.34 (C-1), 97.07 (C-1), 96.61 (C-1), 96.33 (C-1), 96.26 (C-1), 80.94 (C-4^{I}), 77.51–69.24 (7 × C-2, 6 × C-3, 6 × C-4, 7 × C-5), 77.43 (C-3^{I}), 74.71 (C-1'), 62.40 (C-6^{I}), 62.72–62.33 (6 × C-6),

21.12–20.71 (20 × CH_3); MS (EI, *m/z* (rel.%)): 2038.4 (100), 1851.3 (18), 1030.8 (62), 937.8 (10).

Per-*O*-acetyl-6^{I}-*O*-allyl-β-cyclodextrin 2c. The compound was prepared from 6^{I}-*O*-allyl-β-cyclodextrin (8 g, 6.8 mmol). Column chromatography gave the title compound, 13.16 g (96%), as a white powder whose spectral data were in agreement with the published data [1].

References

1. Jindrich, J.; Pitha, J.; Lindberg, B.; Seffers, P.; Harata, K. *Carbohydr. Res.* **1995,** *266,* 75–80. doi:10.1016/0008-6215(94)00251-A
2. Masurier, N.; Estour, F.; Lefèvre, B.; Brasme, B.; Masson, P.; Lafont, O. *Carbohydr. Res.* **2006,** *341,* 935–940. doi:10.1016/j.carres.2006.02.012
3. Jindřich, J.; Tišlerová, I. *J. Org. Chem.* **2005,** *70,* 9054–9055. doi:10.1021/jo051339c
4. Řezanka, M., Eignerová, B., Jindřich, J., Kotora, K. *Eur. J. Org. Chem.* **2010**, 6256–6262. doi: 10.1002/ejoc.201000807
